# Accurate sampling of undisturbed top sediment from grab sampler collected using aluminum tube and stainless-steel containers for shallow and deep-sea applications

**DOI:** 10.1016/j.mex.2025.103213

**Published:** 2025-02-12

**Authors:** Mutsumi Iizuka, Atsuko Amano, Takuya Itaki

**Affiliations:** a The Research Institute of Geology and Geoinformation, Geological Survey of Japan, AIST, Tsukuba Central 7 AIST, 1-1-1 Higashi, Tsukuba, Ibaraki, Japan; b Estuary Research Center, Shimane University, 1060 Nishikawatu-cho, Matsue, Shimane, Japan

**Keywords:** Microplastic, Marine sediment, Grab sampler, A sediment sampling protocol for analyzing microplastics and macroplastics using a K-grab

## Abstract

This study describes a sediment sampling protocol using a Kinoshita-type grab (K-grab) sediment sampler to collect and analyze microplastics (<5 mm) and macroplastics (>5 mm) in marine sediments. During the GB24 geological survey cruise aboard the *Bosei-maru*, 133 surface sediment samples were collected from depths of 20–800 m. The K-grab, equipped with a head-slide weight mechanism, enhanced sampling efficiency across various sediment types. For microplastics, stainless steel containers and J-shaped aluminum tubes minimized contamination while maintaining sample integrity. Macroplastics were separated using a 5 mm mesh and analyzed on board. Method verification confirmed high-spatial-resolution sampling with minimal contamination. These results demonstrate that the K-grab is a reliable tool for microplastic and macroplastic analysis, providing valuable data on plastic pollution in marine sediments.

• This study describes a sediment sampling protocol using a grab sampler to collect and analyze microplastics (<5 mm) and macroplastics (>5 mm) in marine sediments.
• During the survey, 133 surface sediment samples were collected from depths of 20–800 m, with microplastics handled using J-shaped aluminum tubes and stainless steel containers to minimize contamination while maintaining sample integrity.
• Macroplastics were separated using a 5 mm mesh and analyzed on board. Method verification confirmed high-spatial-resolution sampling with minimal contamination.

## Graphical abstract

**Figure:**
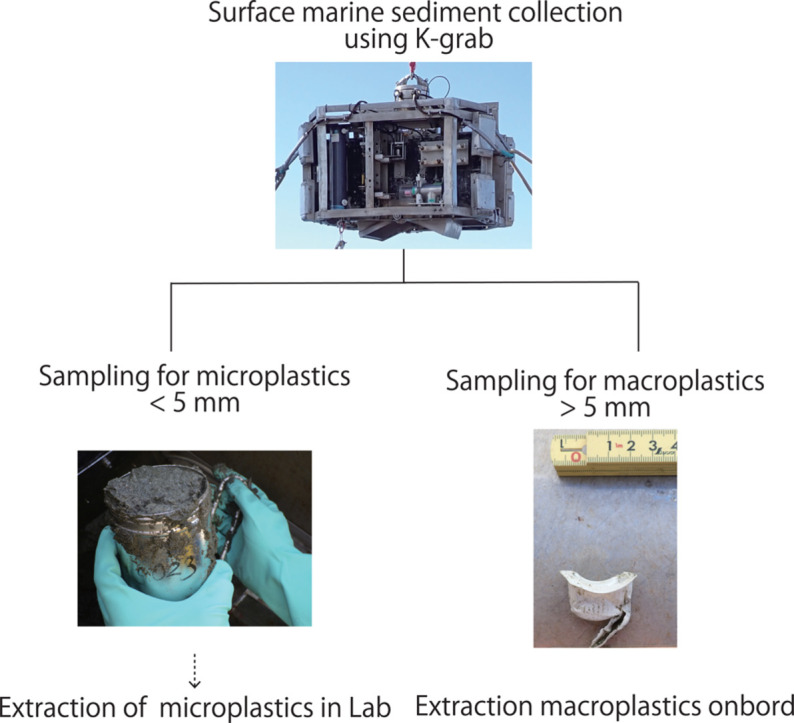

Specifications table

**Table:** 

Subject area:	Environmental Science
More specific subject area:	Sediment plastics
Name of your method:	A sediment sampling protocol for analyzing microplastics and macroplastics using a K-grab
Name and reference of original method:	No name is available to the original method
Resource availability:	Not available

## Background

The release of plastics into the ocean is a pressing environmental issue with severe ecological implications. >10 million tons of plastics enter the ocean annually [[1], [2], [3]], where it undergoes degradation due to the mechanical and photochemical processes accelerated by waves and sunlight, respectively, breaking down into smaller fragments [4,5]. These plastic fragments eventually settle and accumulate in marine sediments [[6], [7], [8]]. However, the transport and deposition mechanisms of plastics vary depending on their size, type, and geographic location, and the processes by which plastics move from continental shelves to deep-sea environments remain poorly understood.

Plastic size differences also influence their ecological impacts. Large plastic debris (>5 mm), such as macroplastics, poses risks of ingestion and entanglement for marine organisms [[9], [10], [11]]. In contrast, smaller plastic particles are more readily ingested and tend to adsorb hazardous substances, raising concerns about their indirect effects through trophic transfer [[12], [13], [14], [15]]. Therefore, understanding the spatial distribution of plastics in marine sediments based on size is crucial for assessing their ecological impact.

Despite this need, no standardized methodology has been established for investigating plastic distribution across continental shelves to deep-sea sediments, making cross-study comparisons difficult. The choice of sediment sampling method depends on water depth and research objectives. Shallow continental shelf sediments are typically collected using grab samplers such as Smith-McIntyre and Van Veen grabs [[16], [17], [18]]. While these samplers are lightweight and easy to operate, they are unsuitable for deep-sea environments and provide limited sample volumes (<5 L in most cases). In contrast, box corers and multiple corers are suitable for deep-sea sampling but are time-consuming and impractical for high-spatial-resolution sampling [19,20]. Furthermore, precise quantification of plastic distribution requires minimizing surface sediment disturbance during sampling. However, existing methods lack a unified approach to prevent disturbance during sampling, complicating accurate plastic quantification.

**Fig. 1:**
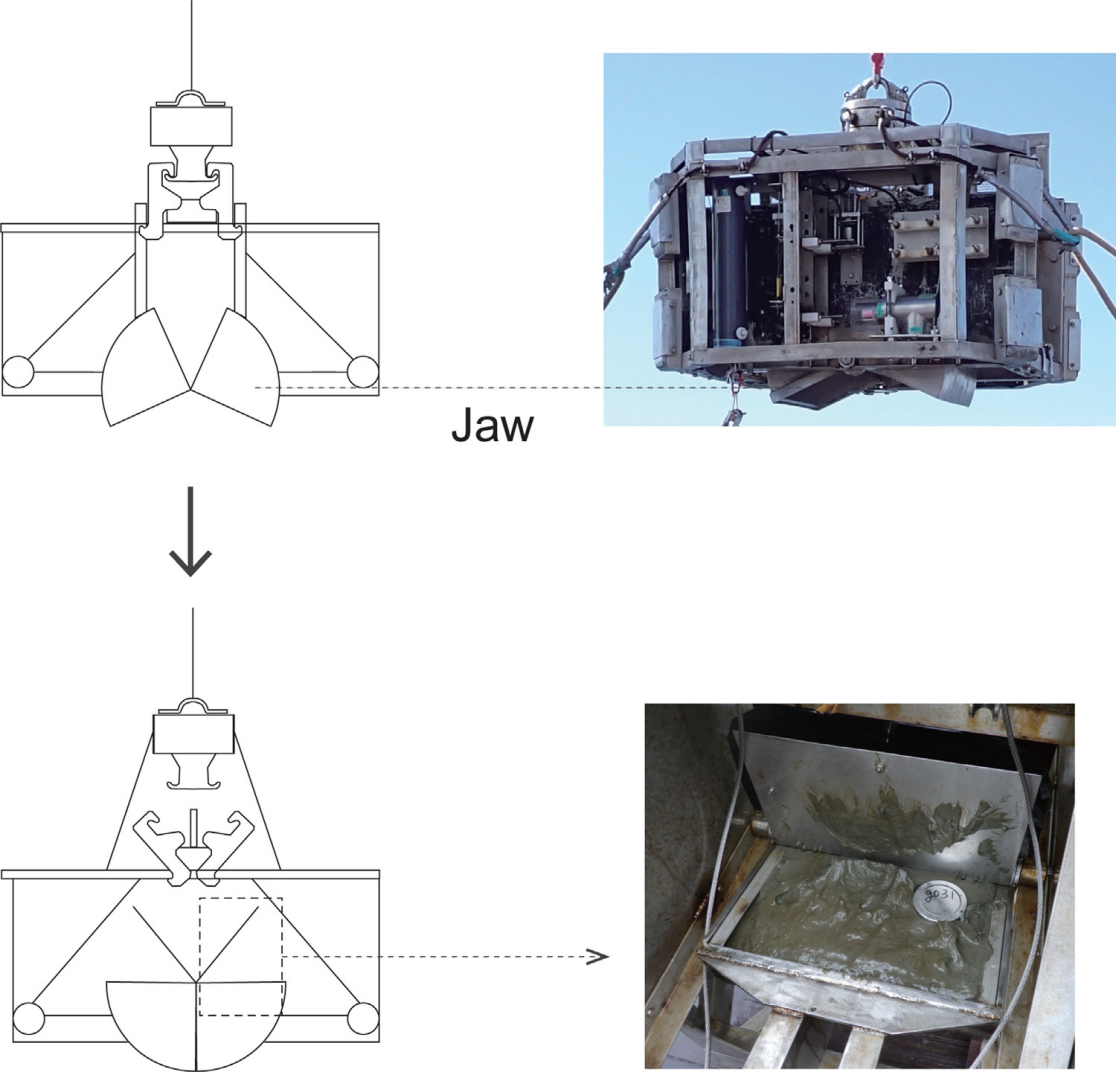
Components of the K-grab Sediment Sampler.

To efficiently assess plastic distribution in marine sediments, a standardized sampling method applicable across both continental shelves and deep-sea environments is necessary. This study addresses this challenge by developing and validating a sediment sampling technique using stainless steel containers and J-shaped tube for Kinoshita-type grab (K-grab) sediment sampler collected sediments (Fig. 1). Compared to conventional methods, this approach minimizes sample disturbance and enables a consistent analysis of sedimentary plastics across various marine environments, facilitating size-based plastic quantification.

## Method details

In this study, the sediment sampling protocol using a K-grab is described for two categories: microplastics (< 5 mm) and macroplastics (> 5 mm). To verify the effectiveness of this method, surface sediment samples were collected using a K-grab during the GB24 geological survey cruise aboard the research vessel “Bosei-maru” of Tokai University. The operation of the K-grab followed the existing procedures of Kinoshita [21], and natural rubber gloves and cotton workwear were worn during sampling operations.

The K-grab can collect up to 39 L of sediment per operation and is entirely made of stainless steel, reducing the risk of plastic contamination. The Smith-McIntyre grabs initiate sediment collection when the base plate slides upward upon seafloor contact, releasing the sampling box from its hook. However, this mechanism can fail in soft seafloor conditions. In contrast, the K-grab used in this study is equipped with a head-slide weight mechanism, which enhances the success rate of sediment collection even in soft sediment conditions and accommodates a wide range of sediments from the continental shelf to the deep sea.

**Fig. 2:**
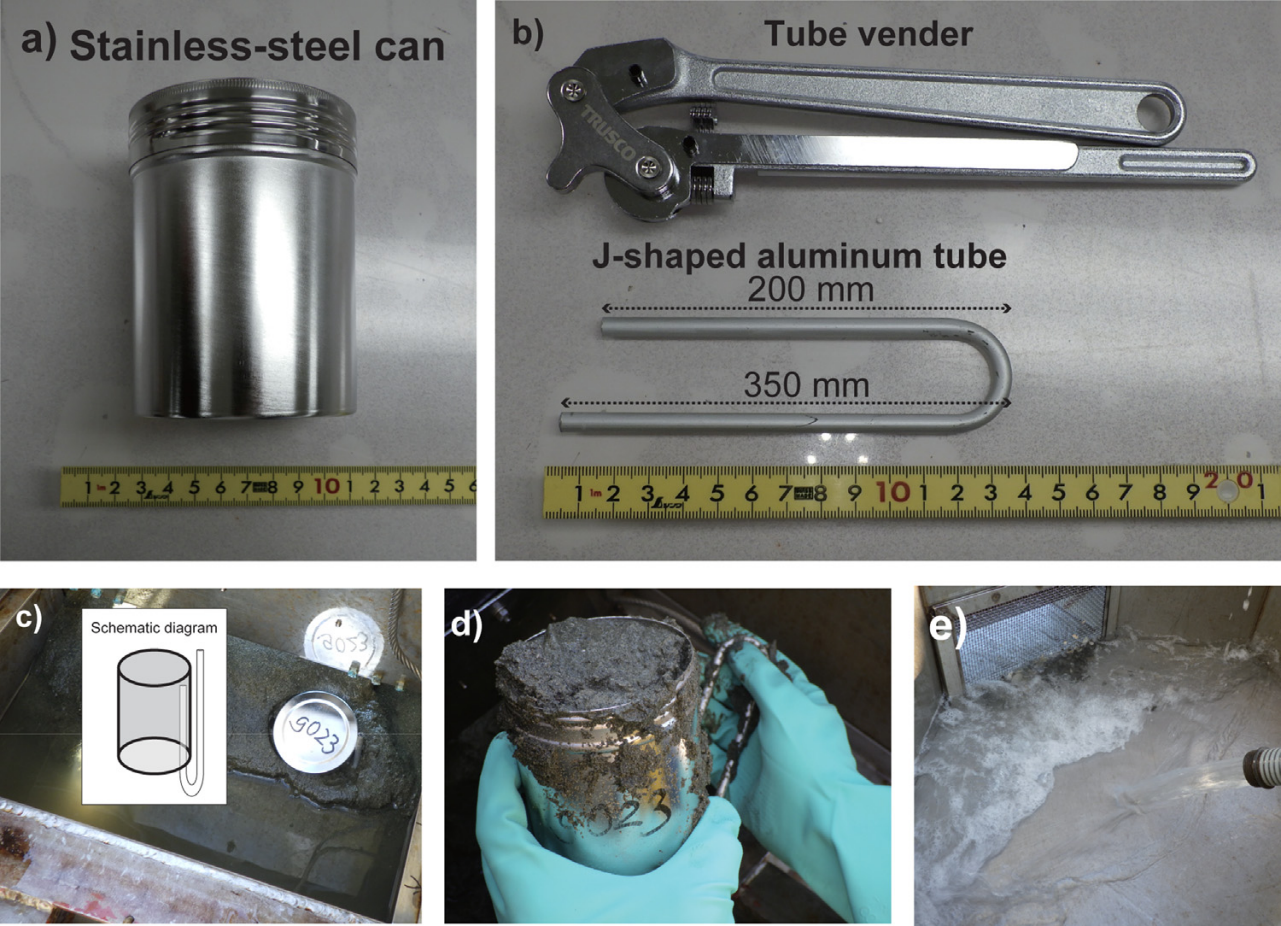
Tools and Steps for sediment sampling for microplastics and macroplastic a) Stainless-steel container, b) Stainless-steel tube and tube bender, c) Inserting the stainless-steel container with the ventilation tube into the sediment, d) Removing the stainless-steel container from the K-grab with sediment, e) Washing the sediment to extract macroplastics.

First, the surface sediment sampling procedure for microplastic analysis is described. Stainless-steel containers and J-shaped aluminum tubes were used for the analysis. A stainless-steel container (85 × 105 × 85 mm, 0.45 L) (Fig. 2a) was selected and cleaned by ultrasonic treatment (15 mins) followed by Milli-Q water rinsing. To ensure ventilation, an aluminum tube (diameter: 0.4 mm) was bent at 180°, with one end measuring 350 mm and the other 200 mm (Fig. 2b). Proper ventilation minimizes physical disturbance of the sediment and helps maintain sample integrity.

After retrieving the K-grab, the stainless-steel container was positioned with its opening facing downward. The shorter end of the aluminum tube was inserted inside the container, while the longer end remained outside (Fig. 2c). Next, the K-grab lid was opened, and the prepared stainless-steel container and aluminum tube were carefully inserted into the sediment (Fig. 2c), ensuring that the sediment did not obstruct the ventilation tube. A stainless-steel scoop was used to remove sediment surrounding the container, and a gloved hand was inserted beneath the container to smoothly extract it without spilling sediment (Fig. 2d). The extracted stainless-steel container was inverted, the sediment surface was leveled with a stainless-steel spatula, and the container edges were wiped with cotton cloth before sealing the lid.

For efficient sampling across multiple sites, a 0.45 L container was used in this study; however, larger sample volumes can be collected by adjusting the container size and tube length as needed. Using stainless steel containers and J-shaped aluminum tubes minimizes plastic contamination while preventing sediment disturbance. The collected samples were transported to the laboratory in the sealed stainless-steel containers for further analysis, following protocols from previous studies (e.g., [[22], [23], [24]]).

To confirm contamination levels during K-grab sampling, a field blank was placed on the upper part of the grab sampler. For instance, a stainless-steel container filled with Milli-Q water was used as a field blank and exposed during sampling. The collected field blanks were returned to the laboratory, where the accumulated microplastics in the sample containers were rinsed, vacuum-filtered, and evaluated [25]. Since airborne plastic concentrations vary with area and wind direction [26,27], field blanks should be collected under different environmental conditions.

Next, the macroplastic sampling procedure is described. Macroplastic analysis requires a larger sediment volume than microplastic analysis. To ensure the collection of macroplastics (> 5 mm), the entire sediment sample obtained by the K-grab was used.

After collecting microplastic samples, a 39 L container was placed beneath the K-grab, and the grab's jaws were opened to transfer all sediment into the container using a stainless-steel scoop. For practical handling, a plastic container was used; however, macroplastics are visible, and contamination was monitored during sampling. The sediment volume in the container was measured and recorded.

The sediment was then transferred to a stainless-steel bucket equipped with a 5 mm mesh and washed with surface seawater (Fig. 2e). To prevent contamination, a mesh filter was attached to the intake hose to ensure the retention of macroplastics larger than 5 mm. After washing, plastic items were identified, recorded, and stored appropriately.

## Method validation

During the GB24 cruise, 133 surface sediment samples were successfully collected over 40 days of investigation, demonstrating the effectiveness of the sampling techniques for acquiring high-spatial-resolution surface sediment data.

The retrieved sediments varied from coarse sand to very fine silt, collected from depths ranging from 20 m to 800 m. The K-grab's collection efficiency depended on grain size, with finer silt-rich sediments yielding up to 39 L, while coarser sediments resulted in a minimum of approximately 5 L. At all sites where sediment depth exceeded 105 mm (the height of the stainless-steel container), a 0.45 L standardized sample was secured. Even at sites with depths below 105 mm, quantitative sampling was possible by measuring sediment height from the top of the container. The J-shaped aluminum tube proved particularly effective for fine-grained sediment collection, ensuring consistent and precise sampling.

The microplastic recovery process was completed within five minutes, minimizing air exposure to less than one minute, effectively reducing contamination risks. Additionally, the stainless-steel container and aluminum tube sampling technique is adaptable to other sediment collection methods, such as the Smith-McIntyre grab and box corers.

The macroplastic sampling process was completed in an average of 15 mins. The only potential contamination source, plastic containers, was thoroughly inspected for fragments, and no contamination was detected. Additionally, during the expedition, macroplastics such as fishing nets and packaging materials were identified at 25 sites, confirming the practical applicability of this approach for shipboard macroplastic analysis.

## Limitations

None.

## Ethics statements

None.

## CRediT authorship contribution statement

**Mutsumi Iizuka:** Conceptualization, Methodology, Writing – original draft. **Atsuko Amano:** Conceptualization, Methodology, Writing – review & editing. **Takuya Itaki:** Conceptualization, Methodology, Writing – review & editing.

## Declaration of competing interest

The authors declare that they have no known competing financial interests or personal relationships that could have appeared to influence the work reported in this paper.

## Data Availability

No data was used for the research described in the article.
